# Two Cases of Endometrial Cancer in Twin Sisters with Myotonic Dystrophy

**DOI:** 10.1155/2016/9737014

**Published:** 2016-08-09

**Authors:** Ezra Y. Koh, Paul J. M. van Kesteren

**Affiliations:** Department of Obstetrics & Gynecology, Onze Lieve Vrouwe Gasthuis, 1091 AC Amsterdam, Netherlands

## Abstract

We describe two cases of endometrial cancer (EC) occurring in nulligravid twin sisters with myotonic dystrophy. Both tested negative for Lynch syndrome and both were treated with laparoscopic hysterectomy with bilateral salpingooophorectomy and adjuvant radiotherapy. Although EC tends to run in families, the diagnosis in itself is not considered sufficient cause for screening or prophylactic measures in close relatives. However, the presence of additional risk factors, such as nulligravidity and myotonic dystrophy in the underlying cases, may call for extra vigilance in first-degree family members.

## 1. Introduction

Endometrial cancer (EC) is currently the most common gynecological malignancy in the developed world [1]. Women who have a first-degree relative with EC are at increased risk of developing EC themselves [2]. In the Swedish Twin Registry, EC concordance/discordance was 1 : 53 among monozygotic twins and 3 : 107 for dizygotic twins [3]. Certain hereditary syndromes, such as Lynch syndrome, are associated with increased risk of developing EC [4]. However, no specific EC genes have been identified as yet. At present, screening of relatives of EC patients is not advocated, because of the relatively low concordance in family members and because of the fact that screening shows little advantage in terms of survival, as symptoms of vaginal bleeding tend to develop early on [5]. In this report, we present two cases of EC occurring in twin sisters, who were both at increased risk of developing EC due to nulligravidity as well as myotonic dystrophy.

## 2. Case Presentation

*Case  1*. In 2013, a 64 year-old lady presented at our clinic with a chief complaint of postmenopausal vaginal bleeding. Her menarche was at age 13, and her last menstrual period was at age 54. She was nulligravid and had a past medical history of chronic obstructive pulmonary disease, hyperthyroidism, and type 1 myotonic dystrophy, though without any clinical signs. No abnormalities were found during physical examination. Her body mass index was 21.5 kg/m^2^. Transvaginal ultrasound revealed a thickened endometrium. Endometrial biopsy showed grade 2 endometrioid adenocarcinoma. Her chest X-ray was unremarkable.

The patient underwent laparoscopic hysterectomy with bilateral salpingooophorectomy (LH-BSO), and her recovery was uneventful. Specimen analysis revealed a grade 3, International Federation of Gynecology and Obstetrics (FIGO) stage Ib endometrioid adenocarcinoma. She was subsequently referred to a tertiary center for adjuvant radiotherapy, where she received 23 fractions of 2 Gy external radiation, followed by 2 fractions of 4.5 Gy high-dose-rate brachytherapy. After 18 months of follow-up, there were no signs of recurrence. Genetic testing for Lynch syndrome was negative. 

*Case  2*. Two years later, the previous patient's twin sister (most likely monozygotic because of phenotypic resemblance, but not confirmed by DNA testing) was also referred to our clinic with postmenopausal vaginal bleeding. She was 66 years old and had a medical history of atrial fibrillation, stroke, subtotal thyroidectomy for multinodular goiter, and type 1 myotonic dystrophy, also without any clinical signs of the disease. Like her sister, she was also nulligravid. Her menarche was at age 13, but her last menstrual cycle was at age 58. Her physical exam was unremarkable. Her body mass index was 22 kg/m^2^. An endometrial thickness of 2.3 cm was measured during transvaginal ultrasound. Endometrial biopsy showed atypical hyperplasia, for which she also underwent LH-BSO. Her postoperative course was uneventful. Pathology revealed grade 2, FIGO stage Ib endometrioid adenocarcinoma. A computed tomography (CT) scan of her chest was unremarkable. This twin, too, was referred for adjuvant radiotherapy (23 sessions of 2 Gy external radiation, followed by 4.5 Gy high-dose-rate brachytherapy).

## 3. Discussion

Our report describes two cases of endometrioid adenocarcinoma occurring in twin sisters. Both patients underwent laparoscopic hysterectomy with bilateral salpingooophorectomy (LH-BSO). In accordance with Dutch guidelines, lymphadenectomy was not performed. Preoperative endometrial biopsy had shown grade 2 EC in patient 1 and atypical hyperplasia in patient 2. Intraoperative imaging and laparoscopic exploration did not reveal any lymph node involvement. Surgical pathology revealed grade 3, FIGO stage Ib EC in patient 1 and grade 2, FIGO stage Ib EC in patient 2. Both patients were subsequently treated with adjuvant radiotherapy.

EC remains the most common gynecological cancer in the developed world, with an incidence of 12.9 per 100,000 women [6]. Given the frequent presentation of this disease, there is a need for establishing risk factors for developing EC, so that at risk patients can be identified. Several endogenous and exogenous risk factors have been found, including parity and age at first and last menses, with the relative risk for nulliparous women developing EC being 3.1 [7]. Hyperestrogenism is also associated with a significantly increased risk of developing EC [8]. Estrogen excess can be caused by hormonal therapy (unopposed estrogens) or by increased endogenous synthesis, for instance, in the events of early menarche, late menopause, or increased aromatization due to obesity or estrogen-secreting tumors. Another risk factor is the use of tamoxifen, and women on tamoxifen who show vaginal bleeding should always be examined to rule out EC. Furthermore, chronic anovulatory conditions, such as polycystic ovarian syndrome, are known to increase the likelihood of developing EC. Both sisters had their menarche at a normal age, did not use any unopposed estrogens, and had a normal body mass index. They were both nulligravid, but patient 2 had longer estrogen exposure due to her later menopause.

Studies have shown a family predisposition to EC in first-degree relatives [7]. Although no specific EC genes have been identified, several genetic syndromes seem to be associated with EC. Current studies show an increased incidence of EC in women who carry the BRCA gene [9]. Another group of patients at increased risk of developing EC are women with hereditary nonpolyposis colorectal cancer, also known as Lynch syndrome [4]. Cowden syndrome is associated with elevated risk [10]. Given the increased risk of EC in patients with Lynch syndrome, both twins were screened for this hereditary condition but tested negative. Further research is needed to investigate the risks and benefits of prophylactic measures in these women.

Although neither twin showed any clinical signs of the disease, both suffered from type 1 myotonic dystrophy, a CTG trinucleotide repeat disorder. This disease is characterized by muscle dysfunction throughout the body in smooth, skeletal, and cardiac muscle. A large Scandinavian survey showed that patients with myotonic dystrophy are at increased risk of developing EC (standardized incidence ratio = 7.6), as well as ovarian cancer, brain cancer, and colon cancer [11]. Overall, patients with myotonic dystrophy have a significantly reduced life expectancy in comparison to the general population [12].

When EC was diagnosed in the first patient, her twin was known to be at increased risk of developing EC because of their kinship, her elevated estrogen exposure (null-gravidity and late menopause), and her history of myotonic dystrophy. When two years later she, too, was diagnosed with EC, the question was raised whether prior screening for EC would have been prudent. The value of screening in such situations has never been studied, so it remains uncertain whether screening would have led to earlier detection and better prognosis in her case. In retrospect, however, extra vigilance may have been warranted.

Recent preliminary studies have demonstrated the value of several biomarkers in screening for EC. Endometrial cancer tissue shows elevated expression of the Ras-related protein Rab-8A, as well as MST1 and PKN1, which could therefore serve as EC biomarkers [13, 14]. Pan-cancer biomarkers have been subject of investigations, too, and may be targets for additional screening in the future [15]. Further, several driver genes have been discovered that could be utilized for more accurate early risk assessment for developing EC [16]. Although these new techniques appear promising, these novel screening modalities are still in early phases of development, and further studies are needed to establish their true value.
